# Electrically stimulated optical spectroscopy of interface defects in wide-bandgap field-effect transistors

**DOI:** 10.1038/s44172-023-00053-8

**Published:** 2023-01-31

**Authors:** Maximilian W. Feil, Hans Reisinger, André Kabakow, Thomas Aichinger, Christian Schleich, Aleksandr Vasilev, Dominic Waldhör, Michael Waltl, Wolfgang Gustin, Tibor Grasser

**Affiliations:** 1 Institute for Microelectronics, TU Wien, Gußhausstraße 27-29/E360, Wien, 1040 Austria; 2 Infineon Technologies AG, Am Campeon 1-15, Neubiberg, 85579 Germany; 3 Infineon Technologies Austria AG, Siemensstraße 2, Villach, 9500 Austria; 4 Christian Doppler Laboratory for Single-Defect Spectroscopy at the Institute for Microelectronics, TU Wien, Gußhausstraße 27-29/E360, Wien, 1040 Austria

**Keywords:** Electrical and electronic engineering, Electronic devices, Fluorescence spectroscopy, Characterization and analytical techniques, Fluorescence spectroscopy

## Abstract

Wide-bandgap semiconductors such as silicon carbide, gallium nitride, and diamond are inherently suitable for high power electronics for example in renewable energy applications and electric vehicles. Despite the high interest, the theoretical limit regarding device performance has not yet been reached for these materials. This is often due to charge trapping in defects at the semiconductor-insulator interface. Here we report a one-to-one correlation between electrically stimulated photon emission and the threshold voltage shift obtained from a fully processed commercial 4H-SiC metal-oxide-semiconductor field-effect power transistor. Based on this observation, we demonstrate that the emission spectrum contains valuable information on the energetic position of the charge transition levels of the responsible interface defects. We etch back the transistor from the reverse side in order to obtain optical access to the interface and record the emitted light. Our method opens up point defect characterization in fully processed transistors after device passivation and processing. This will lead to better understanding and improved processes and techniques, which will ultimately push the performance of these devices closer to the theoretical limit.

## Abstract (web summary)

Maximilian Feil and colleagues report an optical method to characterize interface defects for wide-bandgap transistors. The approach will help to optimize passivation techniques and push the performance of transistors to their theoretical limit.

## Introduction

Wide-bandgap transistors are emerging semiconductor devices for high-voltage electrical energy conversion, such as in photovoltaic inverters, motor drives, or battery charging of electric vehicles^1^. Besides their suitability for applications in quantum photonics^2–6^, these semiconductors feature high thermal robustness^7^, whereby their main advantage over comparable silicon (Si) based devices is the several times higher breakdown voltage, originating from the three times larger wide-bandgap well above 3.0 eV^8–10^. It enables the creation of smaller power devices and hence lower capacitances resulting in increased energy efficiency, particularly, in applications at higher frequencies and elevated temperatures^11–15^. Therefore, such devices are an essential part of the roadmap towards compact and low-loss energy conversion for numerous green technologies leading to long-range electric vehicles and increased efficiency in renewable energy generation.

However, compared to the thoroughly investigated Si/SiO_2_ interface, the interfaces of wide-bandgap semiconductor devices to their insulators exhibit a much higher density of interface defects^16^. For example, with densities up to 4 × 10^12^ cm^−2^ eV^−1^^17^, the 4H-SiC/SiO_2_ interface defect density is about a hundred times higher than that at the Si/SiO_2_ interface, while similar values of 9.9 × 10^11^ cm^−2^ eV^−1^ and 6 × 10^12^ cm^−2^ eV^−1^ have been reported for GaN/AlGaN/SiN and diamond/Al_2_O_3_, respectively^18,19^. Consequently, once an electric potential *V*_GS_ is applied between the gate and source terminals of such a field-effect transistor (FET), these defects can be charged and discharged by exchanging charge carriers with valence and conduction band of the semiconductor via non-radiative multiphonon (NMP) charge transfer reactions^20–22^. This phenomenology leads to a hysteresis as well as long-term drifts in the threshold voltage *V*_th_, known as bias temperature instability (BTI), and to a reduction of the apparent channel mobility^23^. Via its impact on the gate voltage overdrive, a change in the threshold voltage leads to a change in the on-state resistance^24^. This can affect other components of an application circuit.

It is important to realize that the involved defects cover an extremely wide range of time scales from picoseconds to years^24–26^. The fastest defects with time constants up into the milliseconds regime lead to short-term charge trapping and detrapping. This typically results in hysteresis of the threshold voltage that can be observed in the current-voltage characteristic for consecutive up and down sweeps of the gate to source potential (see Supplementary Fig. 1)^17,27^. Slower traps, on the other hand, contribute to BTI. In addition to pre-existing defects, BTI can also contain a quasi-permanent component caused by the creation of new defects^28^. However, hysteresis and BTI are inherently linked by the physical mechanism of fully recoverable charge trapping and detrapping leading to shifts in the threshold voltage ^29^.

While defect-assisted processes are typically of a non-radiative nature, some studies have observed and investigated the light emission from the pn-junction of the semiconductor body diode. Others have also performed optical experiments based on laser excitation^30–36^. In contrast to these well-known approaches, there have been only a few indications that switching a SiC MOSFET electrically from accumulation to inversion or vice versa can lead to light emission via a radiative recombination process^37–40^. By detecting photons through the polysilicon and the SiO_2_ gate dielectric of dedicated test structures, field-effect stimulated radiative recombination was observed in the SiC bulk and via defects at the SiC/SiO_2_ interface, whereby the focus was mainly on time-gating the spectral detection and spatially resolving the spread of the recombining charges.

**Fig. 1:**
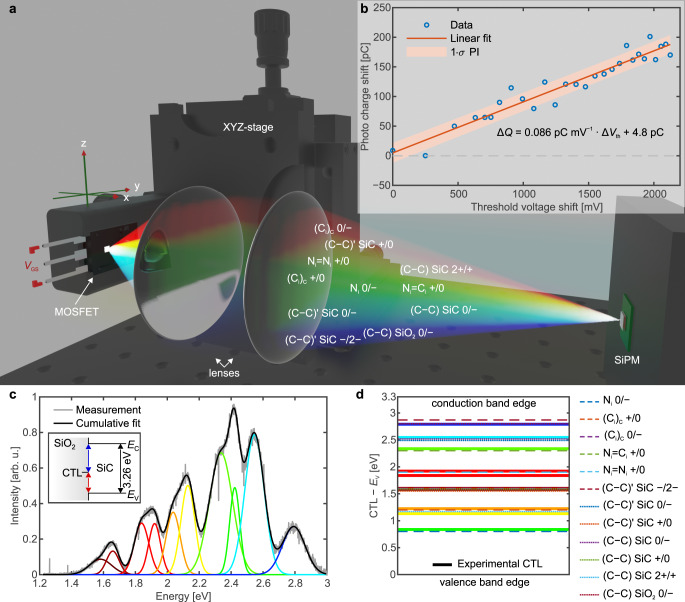
Illustration of measurement setup and main findings. **a** Setup for the detection of the photon emission caused by single gate voltage transients based on a silicon photomultiplier (SiPM). A system of two achromatic lenses images the emitted light from the MOSFET onto the SiPM detector, whereby the alignment of the sample can be performed with an XYZ-stage and a dedicated 3D-printed holder for the discretely packaged device. **b** The photo charge shift versus the threshold voltage shift obtained by the stress scheme illustrated in Fig. 5a. A linear fit and the corresponding 1*σ* prediction interval (PI) indicate a linear relationship between the threshold voltage shift and the photo charge shift with a Pearson correlation coefficient of 0.96. **c** The optical emission spectrum under continuous gate voltage switching. The inset shows the vertically indicated energy differences between a charge transition level (CTL) of a defect and the valence (red arrow) and conduction band (blue arrow and lines) of 4H-SiC. Although there are two transition energies per CTL, note that not all of those transitions are necessarily radiative. **d** Potential assignment of theoretical CTLs from literature studies^46,47^ employing density-functional theory (DFT) simulations to observed experimental CTLs.

**Fig. 2:**
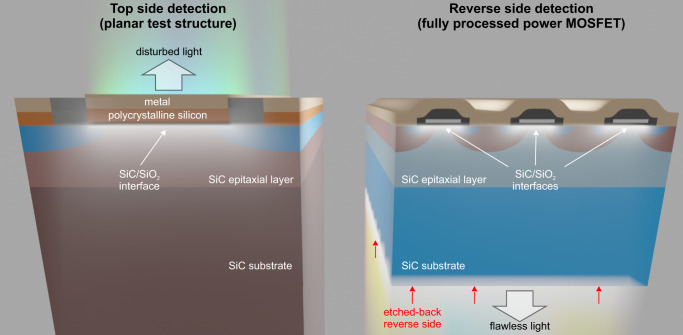
Illustration of optical top side detection and reverse side detection. Previous studies^37–39^ detected through the polysilicon gate contact (left). Not only did this require dedicated test structures, but the absorption of intermediate layers and interfaces also disturbed the extraction of the created photons. In contrast, in our approach, we detect photon emission from the etched-back reverse side (right).

In this article, we investigate field-effect stimulated radiative recombination for the important case of the 4H-SiC/SiO_2_ interface (Fig. 1a), using a novel approach of measuring the light emission from the reverse side of a fully processed commercially available FET (Fig. 2). We begin by showing a one-to-one correlation between photon emission and the threshold voltage shift affecting the device performance (Fig. 1b). This implies that the emitted photons can be used to study the technologically highly relevant threshold voltage shift in-situ during device operation. Furthermore, the observed emission spectrum provides information on the energetic position of the involved defects within the bandgap (Fig. 1c, d). This link to the photon emission paves the way towards in-situ optical characterization of hysteresis and BTI in wide-bandgap semiconductor devices.

In “Emission microscopy”, we investigate the linearity of photon emission with increasing frequency by using emission microscopy and the origin of the emitted light within the active area of the power MOSFET chip. These insights are followed by an investigation in “Bias dependence” on the photon emission during continuous gate switching using a silicon photomultiplier. We managed to directly measure the photon emission of single transitions between accumulation and inversion allowing for temporal separation of recombination events originating from the interaction with the valence and the conduction band. Furthermore, we determine the voltage level dependence and link it to the threshold voltage shift and the capacitance-voltage characteristic of the device. “Emission Spectrum” contains an analysis of the emission spectrum comprising its decomposition into ten single optical transitions at room temperature and a consequent comparison with theoretically calculated charge transition levels. With regard to BTI, we further investigate in “Impact of long-term positive gate bias” the impact of long-term application of a positive gate to source potential. Finally, we discuss the results and present our conclusions in “Discussion” and “Conclusions”.

## Results

### Emission microscopy

First, we employed emission microscopy to visualize the photon emission created during continuous gate switching as observed by Stahlbush et al.^37,38^ and Macfarlane et al.^39^. However, in contrast to their work, we inspected the devices from the reverse side (see Fig. 2), so that the light emitted from the 4H-SiC/SiO_2_ interface only had to pass the 4H-SiC epitaxial layer and the substrate. Contrary to the polysilicon gate, where the photons have to traverse the SiO_2_ and polysilicon layers, this light path is mostly transparent for photons with an energy below the bandgap of 3.26 eV. We expect only minor absorption by n-type 4H-SiC from donor doping states to higher energy levels around 464 nm (2.672 eV)^41^. In contrast to the previously used front-side detection, this technique minimizes the disturbances caused by photon absorption of intermediate layers and interfaces. It allows us to perform our presented experiments on fully processed power FETs, while minimizing any unintended absorption because only the SiC epitaxial layer and SiC substrate lie between the SiC/SiO_2_ interface and photon detection, with both being transparent in the visible spectral range.

**Fig. 3:**
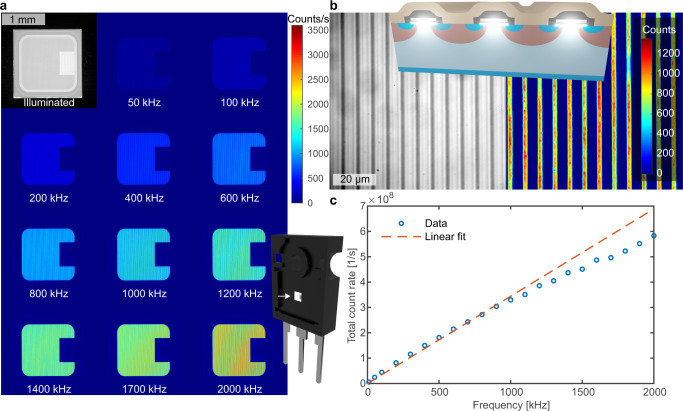
Emission microscopy of 4H-SiC power MOSFET under continuous gate switching. **a** Background-corrected images of the reverse side of the 4H-SiC MOSFET chip using a 5-time magnification objective with an emission microscope. The first image is illuminated to present the chip structure whereas the other images show the photon emission upon gate switching between −10 V and 10 V with various frequencies. **b** An illuminated image and an image of the photon emission as shown in **a**, however with a 100-time magnification objective. **c** Background corrected cumulative photon count rate versus frequency.

We then exposed the MOSFET to a nearly square gate waveform with voltage levels of −10 V and 10 V. Hereby, we varied the frequency between 50 kHz and 2 MHz and integrated the emitted light over a certain time, which was varied between 1 s and 24 s so that the detector did not saturate. Afterwards, we determined the background for each image by calculating the average counts in the upper left 100 × 500 pixels, subtracted it, and normalized the output by the integration time. This resulted in an image of the emission rate of each pixel (see Fig. 3a). The first image shows the illuminated reverse side of the chip. All other images (1024 × 1024 pixels) were obtained in the dark by applying gate voltage signals with varying frequencies and detecting the emitted light. Apparently, the entire active area emits photons, whereby their number increases continuously with increasing frequency. Subtracting the background and changing to a hundred times magnification objective revealed that the emission is localized in the channel underneath the gate insulator (see Fig. 3b). The used power transistor chip consists of several MOSFETs arranged in lines and connected in parallel, which results in long vertical stripes of photon emission from the 4H-SiC/SiO_2_ interface at the channel region (compare to the layout of a power MOSFET in the inset). Finally, summing up the values of all pixels in the background corrected images yields the total count rate in dependence on the frequency (see Fig. 3c). As the emission is caused by switching the MOSFET between accumulation and inversion, in the first approach we basically expected the photon flux to be proportional to the frequency *f*. The slope of such a linear relationship would then reflect the number of photons emitted per gate voltage period. However, this relation would only hold if the number of photons per gate transient from inversion into accumulation and from accumulation into inversion are independent of frequency changes.

1 $$\frac{{{{{{{{\rm{d}}}}}}}}{N}_{{{{{{{{\rm{total}}}}}}}}}}{{{{{{{{\rm{d}}}}}}}}t}=\left({N}_{{{{{{{{\rm{inv-acc}}}}}}}}}+{N}_{{{{{{{{\rm{acc-inv}}}}}}}}}\right)\times f$$

As can be seen in Fig. 3c, this seems indeed to be confirmed for frequencies below 1 MHz as the total detected counts can be well approximated by a linear fit. However, with increasing frequency, the number of detected photons slightly deviates from the linear fit. Such a violation of the linear relation to the frequency is caused by a change in the number of photons emitted by at least one of the two types of transients. This could be due to the *RC* time constant of the MOS capacitor or due to changes in the underlying recombination process. Therefore, it is of particular interest to study the photon emission for the two types of gate voltage transients separately, without the necessity to integrate over several seconds of high-frequency continuous gate switching.

### Bias dependence

Measuring the photon emission of single-gate voltage transients requires a highly sensitive detection unit. Fortunately, the bandgaps of SiC, GaN, and diamond are roughly three to five times larger than the bandgap of silicon, which allows us to establish light detection using a silicon-based photodetector.

**Fig. 4:**
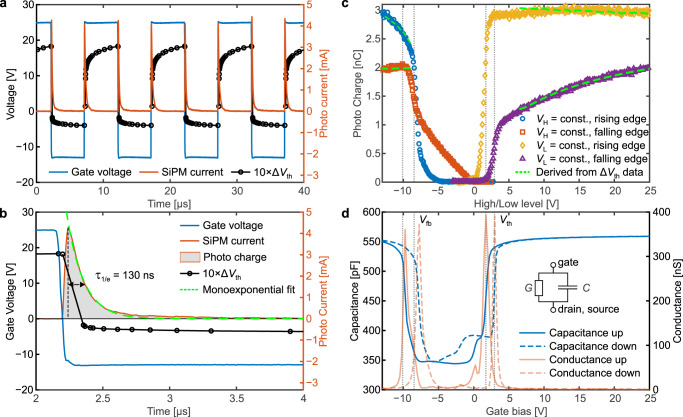
Comparison of optical and electrical measurement results. **a** Continuous gate voltage switching between deep accumulation and inversion and the resulting photocurrent from the SiPM. The threshold voltage shift Δ*V*_th_ during the AC gate signal is shown using a measurement delay time of 1 μs. **b** Magnification of one photo current peak, whereby the integral in time of the peak is proportional to the total charge created at the respective gate voltage transition. A monoexponential fit to the photocurrent decay is indicated. **c** Voltage level dependence of the number of detected photons (photo charge) for rising and falling-edge recombination, while keeping one of the two voltage levels constant. Green dashed lines indicate the relation to the threshold voltage shift Δ*V*_th_ according to equation (4). **d** Gate-source impedance measurement of the 4H-SiC MOSFET at a frequency of 1 kHz interpreted with the equivalent circuit shown in the inset. The capacitance *C* and the conductance *G* are plotted versus the gate bias for an up and a consecutive down sweep. The threshold voltage *V*_th_ and the flat band voltage *V*_fb_ are indicated by vertical lines.

Indeed, the high photodetection efficiency of ~40% and a considerable internal gain of ~5 × 10^6^ of a silicon photomultiplier (SiPM) allows us to detect even single photons. Our experimental arrangement is presented in Fig. 1a, and a detailed description can be found in “Samples and measurements”. This arrangement enabled us to observe the light emission at a single transition from accumulation to inversion or vice versa (see Fig. 4a). By continuously switching the gate voltage of the MOSFET, light emission is detected both at the rising and the falling edge of the gate signal, hinting towards two different types of recombination^39^. Temporally integrating a single SiPM peak yields the released photo charge at the respective gate voltage transient, which is proportional to the number of impinged photons (see Fig. 4b). A monoexponential decay of the SiPM current indicates that most photons are emitted simultaneously, as a monoexponential decay is the intrinsic recovery behavior of a SiPM cell. Light emission at a gate voltage transient is therefore a fast process happening at the gate voltage edge^40^. The first hint of a relationship between the photon emission and hysteresis can be found by comparing the photon emission in Fig. 4 with recent electrically measured findings by Puschkarsky et al.^27,42^. They showed that the threshold voltage shift, as a measure of the number of trapped charges, can easily follow an equally constructed gate voltage signal as the one that triggers photon emission. An analogously executed measurement is shown in Fig. 4. During the application of a positive gate bias, the threshold voltage shift increases rapidly within a fraction of a microsecond. Conversely, the application of the negative gate bias instantly flips the sign of the threshold voltage shift to negative and then decreases even further with time. At the gate voltage transients, fast changes in the threshold voltage shift indicate fast and strong trapping/detrapping of charge carriers. Interestingly, this temporally relates to the emission of photons, observable by the SiPM current.

The possibility of measuring individual emission events of single gate voltage transients allowed us to determine the respective voltage level dependence of both rising and falling-edge emission separately. Transitions from accumulation to inversion (rising edge) and transitions from inversion to accumulation (falling edge) were studied with short double pulses of the gate voltage as shown in Supplementary Fig. 2. In order to facilitate the return of the defects to equilibrium, we used only short 1 μs double pulses. For a particular pair of voltage levels, the SiPM signal was averaged over ten double pulses separated by 1 s, which guaranteed full recovery^43^. The combs of double pulses were in turn separated by 2 s. Either the low level (*V*_L_) or the high level (*V*_H_) of the gate voltage was kept constant at −13 V or 25 V, respectively, while the other voltage level was swept in steps of 0.1 V. Such an approach is similar to the constant base level technique in electrical charge pumping experiments^44^. The photo charge was then determined by integrating the photocurrent peak located at the full gate voltage transient. The observed dependences are presented in Fig. 4c. For the low-level sweep (see the red and blue arrows in Supplementary Fig. 2 and curves in Fig. 4c), both types of recombination show a strong increase around −8 V. Nevertheless, the falling-edge recombination starts to increase from 0 V downwards. Similarly, in the case of the high-level sweep, the emission intensity increases steeply at a certain point around 2 V. For both types of recombination, the onset of emission of the rising-edge recombination is at a lower voltage than the onset of the falling-edge recombination. A comparison of these curves with an impedance measurement of the same type of device strongly suggests that the onsets of photon emission are precisely located around the threshold and flat band voltages (Fig. 4d). In particular, the capacitance and conductance curves can even explain the origin of the shift of the emission onsets by the hysteresis of the device characteristics depending on the direction of the gate voltage sweep^45^. As the emission curves behave in the exact same way irrespective of whether the gate voltage is swept in a positive or negative direction, the observed shift of the photon emission reflects the electrostatic impact of trapped charges at the interface.

Moreover, we conducted the same double-pulse experiment with a short interruption between the two pulses to quickly measure the threshold voltage with a measurement delay of only 1 μs. For defects located at the interface, the threshold voltage shift ${{\Delta }}{V}_{{{{{{{{\rm{th}}}}}}}}}\left(V_{{{{{{{{\rm{GS}}}}}}}}}\right)$ is proportional to the ratio of change in the trapped charge Δ*Q* and the oxide capacitance *C*_ox_. Hence, it is approximately proportional to the change in the number of trapped charges Δ*N*.

2 $${{\Delta }}{V}_{{{{{{{{\rm{th}}}}}}}}}\left(V_{{{{{{{{\rm{GS}}}}}}}}}\right)=-\frac{{{\Delta }}Q}{{C}_{{{{{{{{\rm{ox}}}}}}}}}}\propto {{\Delta }}N$$

However, a measurement of the threshold voltage shift after short gate pulses requires special care and attention. This is because charge trapping and detrapping can happen quasi-instantaneously, within less than the shortest possible measurement delay time upon gate bias changes. In “The relation between threshold voltage shift and photon emission”, we discuss a few general considerations of such experiments that will help to understand the relation between the measured threshold voltage and the photon emission. An overview of the different threshold voltage shift contributions is presented in Supplementary Fig. 3.

The considerations discussed above can be used to understand the relation between measured threshold voltage and photon emission. Following the observation that the threshold voltage shift changes so rapidly at the gate voltage transients, it can be expected that photon emission is caused by the recombination of trapped holes and electrons with the opposite type of charge carrier from the valence or conduction band, respectively. As the number of recombination events is then proportional to the number of involved trapped charges, the absolute threshold voltage shift is proportional to the photo charge *Q*_photo_.

3 $$| {{\Delta }}{V}_{{{{{{{{\rm{th}}}}}}}}}\left(V_{{{{{{{{\rm{GS}}}}}}}}}\right)| \propto {Q}_{{{{{{{{\rm{photo}}}}}}}}}\left(V_{{{{{{{{\rm{GS}}}}}}}}}\right)$$

We can now relate the measured threshold voltage to the measured photo charge. The theory behind this relation is discussed in detail in “The relation between threshold voltage shift and photon emission”. We have to take into account the impact of a parasitic contribution Δ*V*_par_ that effectively increases the measured threshold voltage after a negative gate pulse. For this purpose, we introduced, in addition to the proportionality factor *C*_*s*_, the parameter *V*_0,*s*_. Here, the index *s* indicates whether the measurement was taken after a positive or a negative pulse. *V*_0,*s*_ incorporates the parasitic contribution Δ*V*_par_ after negative pulses into the relation.

4 $${Q}_{{{{{{{{\rm{photo}}}}}}}}}\left(V_{{{{{{{{\rm{GS}}}}}}}}}\right)={C}_{s}| {V}_{{{{{{{{\rm{th}}}}}}}}}\left(V_{{{{{{{{\rm{GS}}}}}}}}}\right)-{V}_{0,s}| ,\,\,\,s\in \left\{+,-\right\}$$

As a result, the relation between ${Q}_{{{{{{{{\rm{photo}}}}}}}}}\left(V_{{{{{{{{\rm{GS}}}}}}}}}\right)$ and $| {{\Delta }}{V}_{{{{{{{{\rm{th}}}}}}}}}\left(V_{{{{{{{{\rm{GS}}}}}}}}}\right)|$ can be broken down into only four parameters. Indeed, the measured data of ${{\Delta }}{V}_{{{{{{{{\rm{th}}}}}}}}}\left(V_{{{{{{{{\rm{GS}}}}}}}}}\right)$ that is transformed by using equation (4) with only four suitable parameters (see Table 1) fit the measured photo charge (see Fig. 4c).

**Table 1:** Parameters of equation (4).

Parameter	Positive pulse (+)	Negative pulse (−)
*C*_*s*_ [nC V^−1^]	0.428	9.499
*V*_0,*s*_ [V]	1.973	4.953

The use of these parameters results in the fit of the voltage-dependent photo charge in Fig. 4c.

The fact that the relation between the threshold voltage shift and photo charge follows equation (3) indicates that the recombination process is related to the same trapped charges that contribute to the threshold voltage shift. In detail, a fraction of charges trapped during the first double-pulse seems to recombine with charge carriers from either the valence or conduction band depending on the direction of the gate voltage transient.

### Emission spectrum

We further analyzed these recombination processes by using a spectrometer to determine the distribution of photon energies. Due to the low light intensity, we had to integrate the incoming photons under continuous gate switching at 1 MHz between 20 V and −10 V over 40 s. As a result, the measured spectrum is a superposition of the spectrum of both rising and falling-edge recombination. The spectrum $I\left(E\right)$ revealed broad band emission between 1.4 eV and 3 eV (see Fig. 1c). As this spectrum covers almost the entire range of visible light, it appears whitish to the human eye. We did not observe any band-to-band recombination around 3.26 eV which is the bandgap energy of 4H-SiC. Hence, the recombination is most likely exclusively via defects within the bandgap of the semiconductor. Most interestingly, this spectrum is composed of well-defined and single transitions and can be fitted with a set of line shape functions. While a set of Lorentzian functions is not sufficient to fit the measurement data, sets of Voigt or Gaussian line shape functions match perfectly. The Voigt function is a convolution of a Lorentzian and a Gaussian function, indicating the broadening of each transition, whereby thermal homogeneous broadening and inhomogeneous broadening due to distributed trap levels are likely to dominate. As can be seen from Fig. 1c, six main peaks as well as four side peaks can be clearly identified in the spectrum. These peaks can be fitted with ten Gaussian functions, leading to

5 $$I\left(E\right)=\mathop{\sum }\limits_{i=1}^{10}{A}_{i}\times \exp \left(-\frac{{\left(E-{p}_{i}\right)}^{2}}{2{\sigma }_{i}^{2}}\right).$$

The parameters of these functions can be found in Table 2.

**Table 2:** Peak parameters of spectral fit.

*p*_*i*_ (eV; nm)	FWHM (meV)	*A*_*i*_	Defect candidate	
1.58; 784	164	0.11	(C–C)’, (C_i_)_C_, (C–C)	VB
1.66; 748	93	0.17	(C–C)’, (C_i_)_C_, (C–C)	CB
1.84; 674	108	0.37	N_i_=N_i_	VB
1.92; 646	88	0.37	N_i_=N_i_	VB
2.04; 609	104	0.45	(C_i_)_C_, (C–C)	CB
2.13; 582	104	0.64	(C_i_)_C_, (C–C)	CB
2.34; 531	172	0.87	N_i_=C_i_	VB
2.42; 512	76	0.62	N_i_	CB
2.55; 487	143	1.00	(C–C)’, (C–C)	VB
2.79; 444	168	0.34	(C–C)’, (C–C)	VB

Defect candidates are taken from Deák et al. ^46^ and Devynck et al. ^47^. A comparison of the associated CTLs can be found in Fig. 1d. The associated charge carrier reservoirs with which the radiative recombination occurs are indicated.

Indeed, extensive theoretical studies employing density-functional theory (DFT) with a hybrid exchange functional have found charge transition levels (CTLs) each of which gives rise to two energies depending on whether recombination is taking place via the valence or the conduction band. These theoretical CTLs closely match our experimental CTLs and give rise to defect candidates that could create the observed spectrum (see Table 2 and Fig. 1d). Please note that the assignment of experimentally observed peaks to certain defect types is, at the moment, not unique. Even though there is, in general, a good agreement between our measured optical spectra and the defect candidates investigated in ref. ^46,47^, it has to be kept in mind that the corresponding theoretical transition energies are deduced from single-particle excitations within the independent Kohn-Sham picture. Indeed, considering lattice vibrations or collective electronic excitations could provide corrections that might explain some partial disagreements. However, including those effects would require the use of more elaborate ab-initio methods such as electron-phonon coupling or solving the Bethe-Salpeter equation (BSE). To the best of our knowledge, such investigations have not yet been conducted for defects at the SiC/SiO_2_ interface. In addition, as the measurement were taken at room temperature, thermal broadening and broadening due to variations in the environment of the respective defect type could also affect the comparison to theoretical results. As already mentioned above, the presence of broadening is well reflected in the fact that the observed spectral transitions do not follow Lorentzian line shapes.

### Impact of long-term positive gate bias

Although a relation between the observed photon emission and the defects involved in BTI can already be inferred from the results presented in the previous sections, a clearer indication is provided in the following.

**Fig. 5:**
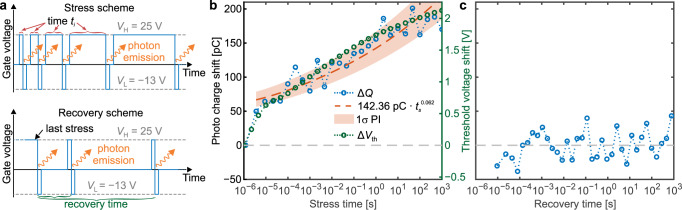
Impact of positive gate stress onto the radiative recombination. **a** Stress (upper schematic) and recovery gate voltage scheme (lower schematic). The time periods at positive bias *t*_i_, and the used low *V*_L_ and high-level *V*_H_ are indicated. **b** The shift of the photo charge (blue circles) versus stress time. A power law (dashed red line) can be fitted analogously to typical threshold voltage drift curves, whereby the 1*σ* prediction interval (PI) is indicated (red area). The threshold voltage shift Δ*V*_th_ (green circles) is associated with the green axis. **c** After all stress phases, the measurement of recovery of the photo charge shift shows that after a single switch into deep accumulation, the involved traps are emptied completely as the emission intensity returns to its initial value.

The MOSFET is exposed to a gate voltage stress scheme illustrated in Fig. 5a. It is composed of logarithmically increasing stress phases interrupted by short measurements of the photon emission. Here, stress means a positive gate voltage applied for a defined period of time. An optical measurement is performed by shortly switching the gate for 1 μs into deep accumulation to trigger the recombination process. After the last stress phase of 10^3^ s, the gate was kept at zero bias, however, with short interruptions by double pulses to trigger and measure the photon emission. We found that with increasing stress time, which is defined by *t*_stress,i_ = *t*_i_ − *t*_0_ (length of the time periods of positive bias application with respect to the very first measurement), the number of detected photons increases with time. Hereby, the stress time is calculated relative to the first stress phase of *t*_0_ = 1 μs. We found that the shift in the measured photo charge Δ*Q* = *Q* − *Q*_0_ with *Q*_0_ = 1.14 nC versus the increase in stress time yields roughly a power law dependence with exponent *n* = 0.062.

6 $${{\Delta }}{Q}_{i}\propto {\left({t}_{i}-{t}_{0}\right)}^{n}$$

A power law dependence of the threshold voltage shift with stress time is a typical signature of BTI, reflecting the increase in the trapped charge. Interestingly, the defects involved in the radiative recombination are discharged completely with a single turn into deep accumulation (see Fig. 5c). Consequently, these defects recover completely and can no longer contribute to a positive threshold voltage shift after a switch into accumulation.

Finally, we confirmed the relation between the observed radiative recombination and BTI by correlating the photo charge shift to the threshold voltage shift. For this purpose, we performed the same measurement as in Fig. 5a, however, we also measured the threshold voltage before the gate was switched to *V*_L_ with a measurement delay time of 1 μs. Another short delay of 10 μs at 0 V gate bias between the *V*_L_-phase and the consecutive stress phase could not be avoided, but its influence on the threshold voltage shift was negligible.

Indeed, the threshold voltage shift behaves almost identically to the photo charge shift, as indicated in Fig. 5b. The threshold voltage shift matches the shape of the photo charge curve even better than the fitted power law. Characteristics such as a strong increase at stress times up to 10^−3^ s and a decrease in slope above 1 s are well reproduced by the curve shape of the threshold voltage shift. As expected, this results in a strong linear correlation between the two quantities (see Fig. 1b) with a near unity Pearson correlation coefficient of 0.96.

## Discussion

Our approach of measuring light from the 4H-SiC/SiO_2_ interface through the reverse side of the chip allows for an almost undisturbed extraction of the emitted light for characterization with a silicon photomultiplier or spectrometer (see Figs. 1–3). The reason for this absence of disturbance is the good transparency of 4H-SiC for the investigated photons located predominantly in the visible spectral range, hence, below the bandgap of 4H-SiC. The comparison between the voltage level dependence of the photon emission and an impedance measurement indicates that recombination is triggered once the device is switched between accumulation and inversion (see Fig. 4). Consequently, the emitted photons stem from the recombination of trapped charges with either holes in the valence band (falling-edge recombination) or electrons in the conduction band (rising-edge recombination). The onsets of photon emission are consistent with the change in device characteristics due to the electrostatic impact of strong short-term charge trapping in 4H-SiC MOSFETs (compare Fig. 4). Although no influence of the etched-back reverse side on the device characteristics could be observed, a minor impact cannot be fully excluded.

**Fig. 6:**
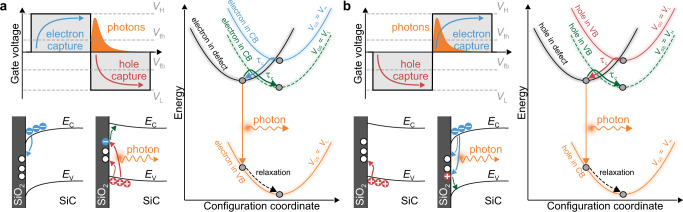
Illustration of underlying mechanisms. Trapping dynamics for **a** a double pulse from inversion into accumulation and **b** for a double pulse from accumulation to inversion. Each panel contains an illustration of the gate voltage pulses versus time (top left), the energy band diagram (bottom left), and the adiabatic potential energy surfaces (right) with indicated trapping/detrapping and recombination processes. In the illustration of the gate voltage pulses, flat band voltage *V*_fb_, threshold voltage *V*_th_, and both low-level *V*_L_ and high-level *V*_H_ of the double pulses are indicated by dashed gray lines. In the energy band diagram, *E*_C_ and *E*_V_ indicate the conduction and valence band edges, respectively. Finally, the NMP transitions are labeled with the associated capture and emission time constants *τ*_c_ and *τ*_e_.

Furthermore, our spectroscopic results are in agreement with theoretical DFT studies, allowing us to assign trap-assisted recombination to interface defects. All in all, these results verify the existence of an additional reaction pathway besides the conventional NMP processes. As already pointed out, this pathway differs depending on whether the device is switched from accumulation to inversion or vice versa (see Fig. 6). When switching from inversion to accumulation, photon emission occurs during the transient of the gate voltage via recombination of trapped electrons with holes from the valence band. The trapping and detrapping time constants *τ*_c_ and *τ*_e_ are still determined by the NMP theory, as NMP relaxation is the dominant process. In contrast to the NMP processes, radiative recombination is a vertical process, most likely followed by phonon scattering leading to a relaxation of the defect state. In contrast, when switching from accumulation to inversion, holes are captured first, followed by recombination with conduction band electrons. It has to be noted that the depicted processes do not represent the full set of possible processes, however, these are likely to dominate over the non-depicted ones. Such a mechanism consistently explains the observation of an increase in recombination with time at positive gate bias, or the increasing absolute gate voltage. The strong linear correlation between the photo charge shift and the threshold voltage shift confirms that a fraction of the defects involved in BTI exhibits radiative pathways under the discussed switching conditions. Interestingly, the involved defects recover completely once the recombination has been triggered. In contrast to the NMP process, the radiative pathway does not exhibit an activation energy barrier (see Fig. 6).

It is possible to measure a single photon signal from the silicon photomultiplier, which allows us to determine a typical number of 300 detected photons. By neglecting any reflection losses at lenses and assuming radiation of photons into a spatial angle of 2*π* from a point source, a typical value for the total number of photons emitted during a gate voltage transition of 2 × 10^4^ can be roughly estimated. This value is then only determined by the geometry of the optics and the photodetection efficiency of the detector. Assuming all the trapped charges are located directly at the interface, a threshold voltage shift of Δ*V*_th_ = 1750 mV would result in roughly 6 × 10^9^ trapped charges. This means that only a fraction of 3 × 10^−6^ of the trapped charges is discharged radiatively. Consequently, the capture and emission time constants must still be dominated by NMP processes. However, the properties of the studied photons allow to investigate the nature of the defects responsible for the hysteresis and BTI in greater detail. Note that besides electrically detected magnetic resonance (EDMR), this is the only technique that provides structural information on the interface of fully processed devices.

Although we expect photon emission in infrared below 1.3 eV, most probably also for silicon devices, the detection is not as simple as in the visible spectral range with a silicon-based photodetector. Wide-bandgap semiconductor devices can particularly profit from this technique because of this simple photon detection in the visible spectral range.

## Conclusions

We have presented an approach to measure field-effect stimulated radiative defect transitions in wide-bandgap devices using the example of the 4H-SiC/SiO_2_ interface in 4H-SiC MOSFETs. It enabled us to temporally separate radiative events originating from defect-assisted recombination with the valence and conduction bands. By closely observing the voltage level dependence of respective recombination, we could link emission properties and charge trapping properties caused by hysteresis and BTI. Our emission spectroscopy could substantiate previous theoretical density-functional theory calculations relating the optical transitions to various electrically active defects. We also observed an increase in recombination when the MOSFET is exposed to long-term positive gate bias. Indeed, there is a strong linear correlation between the threshold voltage shift and the change in the number of emitted photons, indicating an optical relaxation pathway of defects charged during the application of the gate bias.

We anticipate that field-effect stimulated radiative recombination in wide-bandgap semiconductor devices will be a new, unique, and powerful opportunity for investigating the nature of defects at FET interfaces in greater detail by analyzing the emitted photons. Direct access to quantum observables via these photons exceeds the capabilities of any electrical measurement or electron spin resonance experiment and enables the validation of density-functional theory calculations.

## Methods

### Samples and measurements

The MOSFET under test is a commercially available, discretely packaged, planar 4H-SiC DMOSFET. It is operated via the gate and source terminals by a custom-built microcontroller-based setup developed for ultra-fast *V*_th_ readouts and gate signal generation with machine cycle timing precision ^42,48^. Here, the threshold voltage can be measured by connecting the gate terminal to an operational-amplifier-based feedback loop adjusting the gate voltage to a bias featuring 1 mA drain-source current at 0.1 V drain-source voltage. This bias is defined as the threshold voltage and can be measured with 1 μs measurement delay time, which is the period between the end of a particular gate bias and the readout of the threshold voltage.

In order to access the drain side of the 4H-SiC chip, the copper lead frame was first removed in a wet-chemical process with nitric acid. The solder on the reverse side of the chip was then etched away using aqua regia. Finally, the metallization on the reverse side was polished off with diamond paste. Consequently, there is no drain contact remaining. The remaining 4H-SiC has a thickness of ~185 μm.

All optical experiments were conducted in a dark box at room temperature. Our emission microscope is equipped with five and hundred-time magnification objectives and a PM8572A waveform source from Tabor Electronics was used for generating the gate signal. Time-resolved measurements were done with a SiPM, whereby light emitted from the MOSFET is imaged via two achromatic lenses onto the detector (see Supplementary Fig. 4a). The sample was fixed on a 3D-printed holder which was in turn mounted on an XYZ-stage. Furthermore, this setup was protected from ambient radiation by a 3D-printed enclosure. Spectral data were acquired with a fiber-coupled compact CCD spectrometer. The emitted light was collected by an achromatic objective lens and coupled into a multimode fiber using a reflective collimator (see Supplementary Fig. 4b).

Both the SiPM and the CCD spectrometer are silicon-based detectors, which means that the bandgap of silicon limits the range of the detected photons. The spectrometer is able to detect light up to 1000 nm (1.24 eV). Any photon emission above that wavelength, potentially originating from a shallower defect in the bandgap, is not detected.

We further used a Keysight E4990A analyzer for impedance measurements.

### The relation between threshold voltage shift and photon emission

To measure the threshold voltage of a pristine device with a feedback loop (described in “Samples and measurements”), the MOSFET must be switched to the gate bias corresponding to the chosen drain-source threshold current. The time between turning on the feedback loop and measuring the threshold voltage leads to a parasitic threshold voltage shift Δ*V*_par_ > 0. As a result, the measured value ${V}_{{{{{{{{\rm{th}}}}}}}},0}^{s}$ is larger than the real pristine value ${V}_{{{{{{{{\rm{th}}}}}}}},0}^{{{{{{{{\rm{real}}}}}}}}}$.

7 $${V}_{{{{{{{{\rm{th}}}}}}}},0}^{s}={V}_{{{{{{{{\rm{th}}}}}}}},0}^{{{{{{{{\rm{real}}}}}}}}}+{{\Delta }}{V}_{{{{{{{{\rm{par}}}}}}}}}$$

In a crude approximation, we assume the absence of accelerated recovery after the negative pulse during the measurement delay time and negligible additional trapping after a positive pulse ^49^. The latter is justified by the fact that the threshold voltage shift increases linearly on a logarithmic time scale, which makes the additional microsecond negligible. Furthermore, the gate bias during the measurement delay time is lower than it is during the actual positive pulse. This means that negative charges are trapped during the positive pulse and no significant additional trapping takes place afterwards as the defects are already occupied. Consequently, the measured threshold voltage shift after positive (${{\Delta }}{V}_{{{{{{{{\rm{th}}}}}}}}}^{+}$) and negative (${{\Delta }}{V}_{{{{{{{{\rm{th}}}}}}}}}^{-}$) pulses fulfills the following relations.

8 $${V}_{{{{{{{{\rm{th}}}}}}}}}^{+}={V}_{{{{{{{{\rm{th}}}}}}}},0}^{{{{{{{{\rm{real}}}}}}}}}+{{\Delta }}{V}_{{{{{{{{\rm{th}}}}}}}}}^{+}$$

9 $${V}_{{{{{{{{\rm{th}}}}}}}}}^{-}={V}_{{{{{{{{\rm{th}}}}}}}},0}^{{{{{{{{\rm{real}}}}}}}}}+{{\Delta }}{V}_{{{{{{{{\rm{par}}}}}}}}}+{{\Delta }}{V}_{{{{{{{{\rm{th}}}}}}}}}^{-}$$

Here, ${{\Delta }}{V}_{{{{{{{{\rm{th}}}}}}}}}^{+}$ and ${{\Delta }}{V}_{{{{{{{{\rm{th}}}}}}}}}^{-}$ are the true threshold voltage shifts caused by the pulses of respective polarity. It is important to note that the threshold voltage measurement after negative pulses differs from the one after positive pulses as it contains an additional contribution Δ*V*_par_ that arises as well in the measurement of the pristine value.

Relating equation (4) with equations (8) and (9) leads to the following identities for the fitting parameters *V*_0,*m*_.

10 $${V}_{0,+}={V}_{{{{{{{{\rm{th}}}}}}}},0}^{{{{{{{{\rm{real}}}}}}}}}$$

11 $${V}_{0,-}={V}_{{{{{{{{\rm{th}}}}}}}},0}^{{{{{{{{\rm{real}}}}}}}}}+{{\Delta }}{V}_{{{{{{{{\rm{par}}}}}}}}}$$

From the fit parameters listed in Table 1, we can deduct the real threshold voltage ${V}_{{{{{{{{\rm{th}}}}}}}},0}^{{{{{{{{\rm{real}}}}}}}}}=1.973$ V and the parasitic contribution Δ*V*_par_ = 2.98 V. According to equation (7), the pristine value measured with the feedback loop should correspond to the fitting parameter *V*_0,−_. Indeed, a separate measurement results in ${V}_{{{{{{{{\rm{th}}}}}}}},0}^{s}=4.996$ V, which means the fit parameter deviates <1% from the measured value. Additionally, ${V}_{{{{{{{{\rm{th}}}}}}}},0}^{{{{{{{{\rm{real}}}}}}}}}=1.973$ V coincides well with the CV measurement in Fig. 4d. Overall, this suggests the validity of the above considerations.

### Supplementary information

Supplementary Information

## Data Availability

The data that support the findings of this study are available from the corresponding author upon reasonable request.
