# Supplementary material for: Electrically stimulated optical spectroscopy of interface defects in wide-bandgap field-effect transistors
Source: Commun Eng. 2023 Jan 31;2:5. doi: 10.1038/s44172-023-00053-8 (PMC10955897; doi:10.1038/s44172-023-00053-8)
Supplement: Supplementary file 1 — Supplementary Information [file 44172_2023_53_MOESM1_ESM.pdf]

Supplementary Information
Electrically Stimulated Optical Spectroscopy of Interface Defects in Wide-Bandgap Field-Effect Transistors

Maximilian W. Feil[1,2], Hans Reisinger[2], André Kabakow[2], Thomas Aichinger[3], Christian Schleich[4], Aleksandr Vasilev[4], Dominic Waldhör[1], Michael Waltl[4], Wolfgang Gustin[2] and Tibor Grasser[1*]

[1]Institute for Microelectronics, TU Wien, Gußhausstraße 27-29/E360, Wien, 1040, Austria
[2]Infineon Technologies AG, Am Campeon 1-15, Neubiberg, 85579, Germany
[3]Infineon Technologies Austria AG, Siemensstraße 2, Villach, 9500, Austria
[4]Christian Doppler Laboratory for Single-Defect Spectroscopy at the Institute for Microelectronics, TU Wien, Gußhausstraße 27-29/E360, Wien, 1040, Austria
[*]Corresponding author. E-mail: grasser@iue.tuwien.ac.at

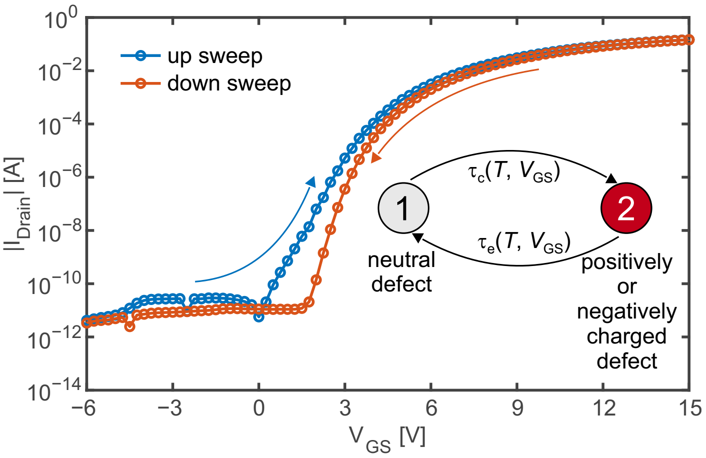

Supplementary Fig. 1: **Up and down current-voltage characteristics of the 4H-SiC power MOSFET** The drain-source current of the investigated SiC MOSFET during slow consecutive up and down sweeps (in 0.25 V steps of the gate voltage V_{GS} with an integration time of 200 ms) of the gate voltage between −15 V and 15 V at a drain-source bias of 0.1 V. The short-term charge trapping/detrapping in defects leading to the observed difference between the up and down sweeps is the so-called hysteresis. The inset schematically shows two defect states 1 and 2, whereby first-order transitions lead to a change in the trapped charge, causing a shift in the threshold voltage. The two processes are characterized by their temperature (T) and gate bias (V_{GS}) dependent capture (τ_c) and emission (τ_e) time constants [1], [2], [3].

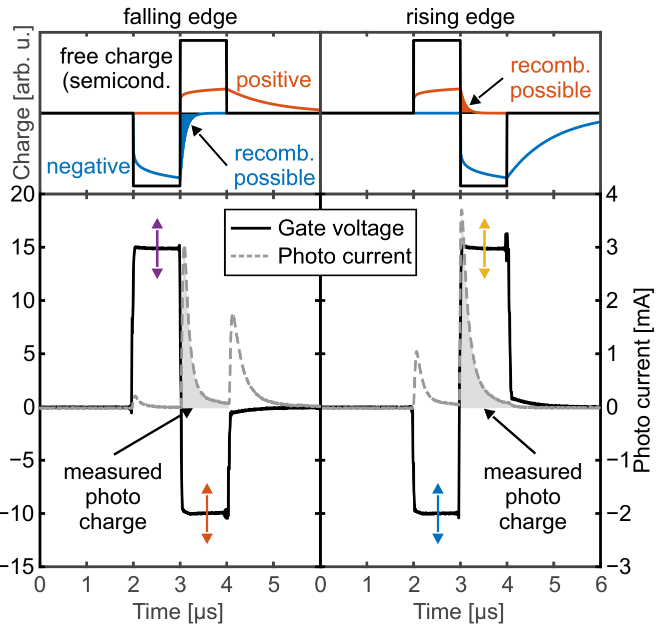

Supplementary Fig. 2: **Measurement scheme for the voltage level dependence** Short, 1 µs-long double pulses are used to study the voltage level dependence of the radiative recombination. The grey area indicates the measured photo charge. Emission peaks appearing during the first pulse are caused due to the repetitive measurement scheme. Hence, some charges trapped during the previous measurement recombine at the edge of the first pulse. We assume that the emission peak at the rising edge of the second pulse, in the left plot, is assigned to the overlap regions of the highly doped source contacts and the gate oxide, where free electrons are available already at 0 V. The upper plots schematically illustrate the amount of trapped charge, either positive or negative, and the free charge in the semiconductor with which the trapped charge can recombine. A temporal overlap between trapped and free charge of opposite polarity allows the recombination process.

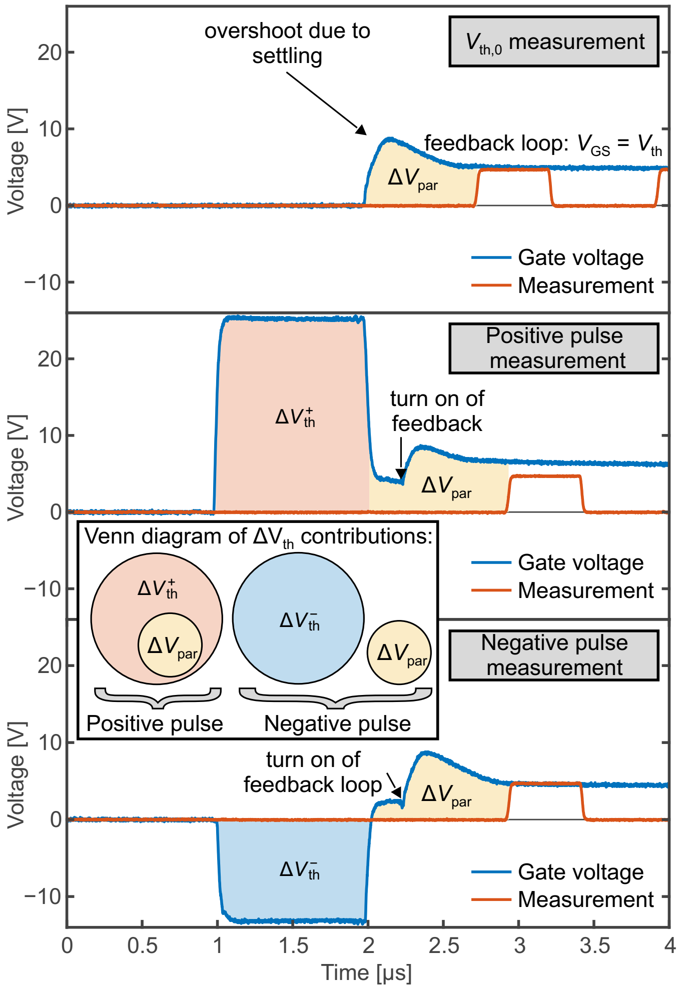

Supplementary Fig. 3: **Illustration of different contributions to the threshold voltage upon different types of measurements with a feedback loop** The upper plot shows a typical initial threshold voltage measurement ($V^s_{\mathrm{th},0}$ measurement). The turn on of the feedback loop leads to a short overshoot of the gate voltage after which it settles to the threshold voltage of the device, which is then measured at the rising edge of the trigger signal. The non-zero gate voltage during the time till the measurement is triggered leads to unintended trapping of negative charges, leading to a change in the threshold voltage ΔV_{par}. In the central plot, a typical measurement after a positive $25\,\mathrm{V}$ $1\,\mathrm{\mu s}$ long pulse is shown. The pulse itself leads to the trapping of negative charges resulting in a threshold voltage shift ΔV^+_{th}. In contrast to the $V^s_{\mathrm{th},0}$ measurement, there is no significant additional trapping of charges after the end of the positive pulse as those charges have already been trapped during the positive pulse. The lower plot shows the same for a threshold voltage measurement after a negative $-13\,\mathrm{V}$ pulse. Here, the important difference from the positive pulse is that after the trapping of positive charges during the negative pulse, additional negative charges are trapped until the threshold voltage measurement is triggered. In consequence, ΔV_{par} appears only in the $V^s_{\mathrm{th},0}$ measurement and the negative pulse measurement. This is illustrated in the inset by a Venn diagram.

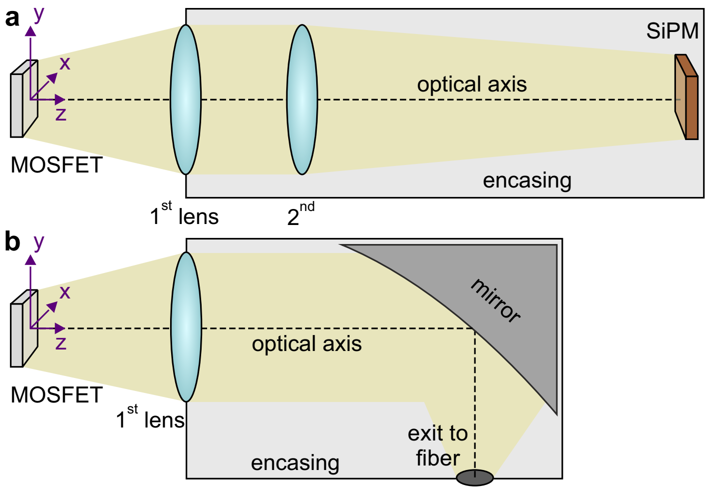

Supplementary Fig. 4: **Illustration of optical measurement setups a)** Setup for the time-resolved detection of the photon emission caused by single gate voltage transients based on a silicon photomultiplier (SiPM). A system of two achromatic lenses images the emitted light from the MOSFET onto the SiPM detector, whereby the alignment of the sample can be performed with an XYZ-stage and a dedicated holder for the discretely packaged device. **b)** Setup for collecting the emitted light during continuous gate switching for spectral analysis. By coupling the emitted light via an achromatic lens with a reflective collimator into a multi-mode fiber, the light can be analysed via a CCD spectrometer.

SUPPLEMENTARY REFERENCES

[1] Grasser, T. Stochastic charge trapping in oxides: From random telegraph noise to bias temperature instabilities. *Microelectronics Reliability* **52** (1), 39–70 (Jan. 2012). https://doi.org/10.1016/j.microrel.2011.09.002 .

[2] Schleich, C. *et al.* Physical Modeling of Charge Trapping in 4H-SiC DMOSFET Technologies. *IEEE Transactions on Electron Devices* **68** (8), 4016–4021 (Aug. 2021). https://doi.org/10.1109/TED.2021.3092295 .

[3] Vasilev, A. *et al.* TCAD modeling of temperature activation of the hysteresis characteristics of lateral 4H-SiC MOSFETs. *IEEE Transactions on Electron Devices* **69** (6), 3290–3295 (June 2022). https://doi.org/10.1109/TED.2022.3166123 .
